# Linking Contact Behavior and Droplet Patterns to Dynamically Model Indoor Respiratory Infections Among Schoolchildren

**DOI:** 10.2188/jea.JE20120031

**Published:** 2013-07-05

**Authors:** Shu-Han You, Szu-Chieh Chen, Chien-Hua Wang, Chung-Min Liao

**Affiliations:** 1 Department of Bioenvironmental Systems Engineering, National Taiwan University, Taipei, Taiwan, ROC; 2 Department of Public Health, Chung Shan Medical University, Taichung, Taiwan, ROC; 3 Department of Family and Community Medicine, Chung Shan Medical University Hospital, Taichung, Taiwan, ROC

**Keywords:** airborne infectious droplets, indoor aerosol transmission, indoor air quality, modeling, influenza

## Abstract

**Background:**

We used the results of a contact behavior survey in conjunction with droplet pattern measurement to investigate the indoor population transmission dynamics of respiratory infections.

**Methods:**

A total of 404 questionnaires on all contact behaviors were distributed to junior high school students. Droplet number concentration and size distribution generated by coughing and talking were measured by droplet experimentation. A deterministic susceptible–exposed–infected–recovery (SEIR) model was used to simulate the indoor transmission dynamics of influenza infection among schoolchildren.

**Results:**

Results indicated that the average contact rates ranged from 9.44 to 11.18 person^−1^ day^−1^ for grades 7 to 9. We showed that total median droplet number concentrations were 9.01 × 10^7^ and 8.23 × 10^7^ droplets per cubic meter for coughing and talking, respectively. Population dynamic simulations indicated that the size-dependent median number of droplets per person resulted in a maximum of 8 and 10 infected persons on day 4, respectively, for talking and coughing activities.

**Conclusions:**

Human contact behavior and airborne droplet characteristics may substantially change predicted indoor population transmission dynamics of influenza infection.

## INTRODUCTION

Among airborne infectious diseases, pandemic and seasonal influenza are highly prominent worldwide. One of the most obvious features of an infectious agent is its ability to be transmitted from person to person. Indeed, patterns of human contact in a host population determine who is at a high risk of contracting an infection.^1^ Stilianakis and Drossinos^2^ maintained that the rate at which a susceptible individual becomes an infectious individual could be estimated by multiplying 2 factors: (1) the contact rate of a susceptible individual to a droplet exhaled by an infectious individual and (2) the probability that contact with an exhaled droplet results in successful transmission to a susceptible individual. However, precise investigations of transmission parameters are relatively sparse. Our study used a questionnaire to survey contact patterns of highly transmissible populations, as well as simple measurements to characterize droplet sizes and numbers in an indoor environment. An infectious disease transmission model was also used to investigate dynamics, after incorporating the measurement data.

Human behavior and activities are linked to a number of processes that introduce infectious droplets into indoor air. Human activities such as coughing and sneezing will generate respiratory droplets with different characteristics. Specifically, it was previously shown that droplet generation and size were key determinants of pathogen carriage, aerosolizing, and transmission.^3^^–^^6^

Early studies evaluated respiratory droplets by means of solid impaction onto a glass slide, followed by microscopic characterization in test participants.^7^^–^^9^ An extension of this method used food dye in the mouth to distinguish droplet deposition.^3^^,^^7^ Recently, optical technology (namely, an optical droplet counter) was used to test droplet size distribution and concentration number for human respiratory activities such as breathing, talking, sneezing, and coughing.^3^^,^^5^^,^^9^^–^^12^

Duguid^7^ reported that a respiratory droplet had a lognormal distribution with a geometric mean (GM) of 14 µm and a geometric standard deviation (GSD) of 2.6 for coughing and a GM of 8.1 µm and a GSD of 2.3 for sneezing. However, Louden and Roberts^8^ found that the estimated lognormal parameters were a GM of 12 µm and a GSD of 8.4 for coughing. More recently, Papineni and Rosenthal^9^ found expired bioaerosol droplets (in nose and mouth breathing, coughing, and talking) to be less than 2 µm in size, with no droplets larger than 8 µm. Chao et al^12^ found that the GM of droplets was 13.5 µm from coughing and 16.0 µm from speaking (participants counted numbers from 1–100). Their research also indicated that the estimated droplet concentrations ranged from 2.4 to 5.2 cm^−3^ per cough and from 0.004 to 0.223 cm^−3^ for speaking.

In light of mathematical modeling, previous research on effective contact rates involved studying social mixing patterns relevant to infectious diseases, leading to development of transmission models.^1^^,^^13^^–^^15^ Different investigative methods and questionnaire tools have also been discussed and compared, including self-evaluation and diary-based data collection methods through a web-based interface.^14^ Hand-held electronic diaries (PDA)^15^ have also been utilized. The timeliness, accuracy, and completeness of contact investigations are key factors in conducting social contact estimations.

Contact patterns are strongly associated with age. High rates of influenza transmission are seen among school-aged children and teenagers in particular. Thus, it is important to estimate age-specific transmission parameters for respiratory infectious agents.^16^^,^^17^ Wallinga et al^16^ suggested that school-aged children and young adults have the highest incidence of infection and that they contribute most to further spread of infections during the initial phase of an emerging respiratory epidemic within a completely susceptible population. Investigation of age groups for contact included adults or young adults, elementary school students, and those younger than 11 years.^18^^,^^19^

The aim of the present research was to investigate the indoor dynamics of respiratory infectious disease by linking contact behavior and droplet patterns with transmission in schoolchildren. A questionnaire survey was used to analyze the daily number of contacts among schoolchildren. A simple measurement for droplet sizes and numbers was also recorded. Finally, these measurement data were incorporated into an infectious disease model that simulated population transmission dynamics.

## METHODS

### Investigation of contacts among schoolchildren

This study was conducted in a junior high school in Jhongli City, Taiyuan County, Taiwan, in March 2010. Two classes in each grade were selected to report their contacts on different days of the week. Each participant was asked to complete 2 questionnaires: 1 on a randomly assigned weekday and 1 on a randomly assigned weekend day.

A contact was defined as a 2-way conversation (at a distance that did not require raising the voice) in which at least 3 words were spoken by each party and in which there was no physical barrier between the 2 parties (such as security screens).^13^ The conversation distance was less than 1 m.^20^^,^^21^ More detailed descriptions of this survey are provided in Chen et al.^22^

### Droplet measurement and sampling design

To estimate the concentration number and size distribution generated by coughing and talking, respiratory droplet experiments were conducted among 10 nonsmoking participants: 6 healthy participants and 4 participants with influenza-like symptoms. The participants were aged from 20 to 30 years and were recruited from a cohort of Chung-Shan Medical University students. The coughing and talking experiments were done in the morning and afternoon for all participants and on all sampling dates. Before the experiment, all participants gave written informed consent to take part in the study.

**Figure 1.**
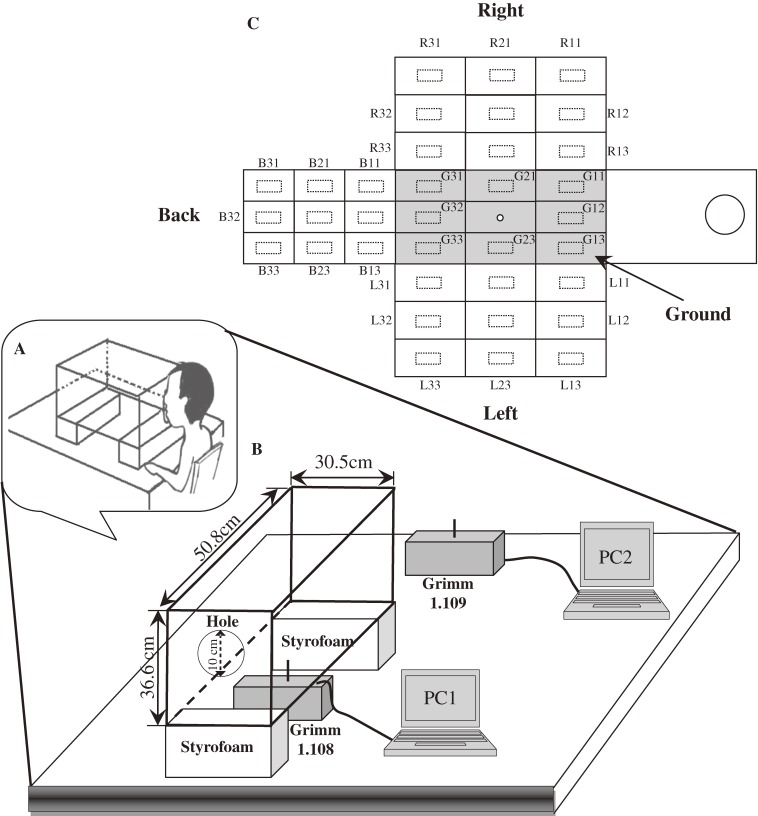
Schematic of expiratory droplet investigation setup. (A) Two dust monitors (Grimm 1.108 and Grimm 1.109) were used to estimate droplet concentration number. An entry hole (diameter, 100 mm) was cut at about two-thirds of the height of the front wall to allow for respiratory droplet expulsion (B) from the participant’s mouth. (C) A total of 35 slides were numbered and attached to the back, left, right, and floor of the sampling box before each test of expiratory activities (ie, talking and coughing).

A schematic of the experimental design is shown in Figure 1. The sampling method and experimental design were partly adopted from Xie et al^3^ and Loudon and Roberts.^8^ A small air-tight box was constructed from Perspex (interior dimensions, 36.6 cm × 50.8 cm × 30.5 cm)^3^^,^^8^ and placed in a clean room (dimensions, 5.61 m × 3.33 m × 2.43 m) equipped with a central filtration system. An entry hole (diameter, 100 mm) was cut at approximately two-thirds the height of the front wall to permit respiratory droplet expulsion from the participant’s mouth (Figure 1A and 1B). Figure 1B shows a schematic of the participant and sampling hole.

A 15-channel dust monitor (Grimm 1.108, Germany) was used for real-time size measurement of exhaled droplets from 0.3 to 20 µm in the sampling box. The sampling flow rate was 1.2 L min^−1^. To monitor droplet concentration in the clean room, a dust monitor (Grimm 1.109, Germany) capable of detecting droplet sizes from 0.22 to 32 µm was placed at a distance of approximately 50 cm from the box. Temperature and relative humidity in the box were also recorded for each participant before and after the experiments. The droplets were collected on microscope glass slides of standard size (76 × 26 mm). A total of 35 slides were numbered and attached to the back, left, right, and floor of the box before each test for each expiratory activity (talking and coughing; Figure 1C).

### Experimental procedure and data collection

Before each experiment, all slides and the sampling box were cleaned 3 times with 75% alcohol and then 4 times with distilled water. After waiting for the slides and box to dry, 35 slides were loaded into the 4 walls inside the sampling box. When the experiments began, the dust monitors (Grimm 1.108 and Grimm 1.109) inside and outside the box were turned on to monitor droplet concentrations simultaneously. Fifteen minutes after activation of the monitors, each participant was asked to enter the clean room, sit on the chair in front of the box, and wait for another 15 minutes. Then, the subject performed the planned expiratory activity (talking or coughing) into the box through the entry hole.

During the talking activity, the subject counted from 1 to 20. During the coughing activity, the subject coughed loudly and slowly for 20 repetitions. To control the speed of each activity, we designed a PowerPoint slide that guided the participant to produce 1 talk/cough behavior in 3 seconds. The exhaled activities lasted for 1 minute (20 times × 3 seconds = 1 minute). As soon as this was completed, the participant was asked to wait for 15 minutes and then leave the clean room. After another 15 minutes, the dust monitors were turned off to reduce the effects of opening and closing the door, thus maintaining a steady environmental droplet concentration. Then, the glass slides were collected in the sampling box. Finally, the sampling box was cleaned for the next subject.

To stain the saliva before the expiratory activities were performed, participants were asked to gargle 2 or 3 times using a dye solution. Powdered red food dye was dissolved in distilled water to reach a concentration of 3 ppm. The large droplets collected from glass slides were observed and counted with a microscope (Nikon SMZ645, objective lens 5×, ocular lens 10×). A Neubauer counting chamber was used to count droplet concentrations on the glass slides.

### Transmission rate quantification

The rate at which a susceptible individual becomes an infectious individual can be estimated by multiplying 2 factors: (1) the contact rate of a susceptible individual to a droplet exhaled by an infectious individual ($\tilde{C}$) and (2) the probability that contact with an exhaled droplet results in successful transmission to a susceptible individual (${\tilde{P}}_{d}$). We assumed that each infected person carried equally infectious droplets. These released droplets were homogeneously mixed with susceptible and infected populations (Stilianakis and Drossinos, 2010).^2^ Thus, all susceptible individuals have an equal probability of being infected. In addition, these released droplets could carry pathogens and lead to successful transmission when susceptible individuals inhale pathogen-carrying droplets.

To quantify the contact rate of a susceptible individual with a droplet ($\tilde{C}$), several parameters should be considered, including number of contacts, inhalation rate, and droplet volumes. Hence, $\tilde{C}$ can be described by the following equation:

$$\tilde{C}=\frac{cB\tau }{V_{\text{cl}}N}\delta t,$$ (1)

where *c* is the average number of susceptible contacts with any individual (day^−1^) during unit time *δt*. This estimation was adopted from investigation of schoolchildren contacts. *B* is the inhalation rate of a susceptible person, expressed as volume of air per unit of time (m^3^ day^−1^), *τ* is the duration of contact between the susceptible and the infected (day), and *V*_cl_ is the personally generated droplet volume (m^3^). *N* is the total population.

Regarding the other factors, to quantify the probability that contact with an exhaled droplet results in successful transmission to a susceptible individual, (${\tilde{P}}_{d}$). ${\tilde{P}}_{d}$ can be described by the following equation:

$${\tilde{P}}_{d}=P_{d}N_{d}q_{\text{dep}},$$ (2)

where *P*_d_ is the probability of transmission per inhaled pathogen, *N*_d_ is the number of pathogens in a droplet of diameter *d*, and *q*_dep_ is the probability of deposition in the human respiratory tract per pathogen. This study classified droplet diameter, *d*, as *d*_1_ = 4 µm and *d*_2_ = 4 to 8 µm for different droplet behaviors. In conclusion, transmission rate can be expressed as

$$\beta =\tilde{C}\times {\tilde{P}}_{d}.$$ (3)

### Susceptible–exposed–infected–recovery model

We used a deterministic susceptible–exposed–infected–recovery (SEIR) model to simulate the indoor transmission dynamics of influenza infection among schoolchildren. The SEIR model provides a basic description of population transmission dynamics by using a simple parameterized set of ordinary differential equations, namely:

$$\frac{dS}{dt}=-\beta D(t)S(t),$$ (4)

$$\frac{dE}{dt}=\beta D(t)S(t)-\sigma E(t),$$ (5)

$$\frac{dI}{dt}=\sigma E(t)-\gamma I(t),$$ (6)

$$\frac{dR}{dt}=\gamma I(t),$$ (7)

N(t)=S(t)+E(t)+I(t)+R(t), (8)

where *N*(*t*), *S*(*t*), *E*(*t*), *I*(*t*), and *R*(*t*) represent the total number of schoolchildren, and the numbers of susceptible, exposed, infected, and recovered schoolchildren populations at time *t*, respectively; *σ* is the rate at which an exposed individual becomes infectious per unit of time (equal to 0.333 day^−1^),^23^ and *γ* is the rate at which an infectious individual recovers per unit of time (equal to 0.2 day^−1^).^24^

In addition, to combine human contact behavior with droplet pattern, the dynamic of droplets ($\frac{dD}{dt}$) is decided by the droplet production rate and removal mechanisms. The droplet removal mechanism includes settling and inactivation. *D* is the total number of droplets per unit of time and can be described as:

$$\frac{dD}{dt}=kI(t)-\nu D(t),$$ (9)

where *k* is the droplet production rate (day^−1^); *ν* is the droplet removal rate (day^−1^). Here, we assumed that the population size of schoolchildren was *N* = 34 individuals. The initial condition (*t* = 0) for talking and coughing modeling was 25, 8, 1, and 0 for *S*(*t*), *E*(*t*), *I*(*t*), and *R*(*t*), respectively.

## RESULTS

### Daily number of contacts

A total of 202 participants were included in this survey, and the basic sociodemographic characteristics of the participants were described in Chen et al.^22^ Nonresponders comprised participants with unrecorded (9.6%) or uncompleted questionnaires (12.1%) and those who were not paired or were missing data for a workday or weekend day (10.3%).

**Figure 2.**
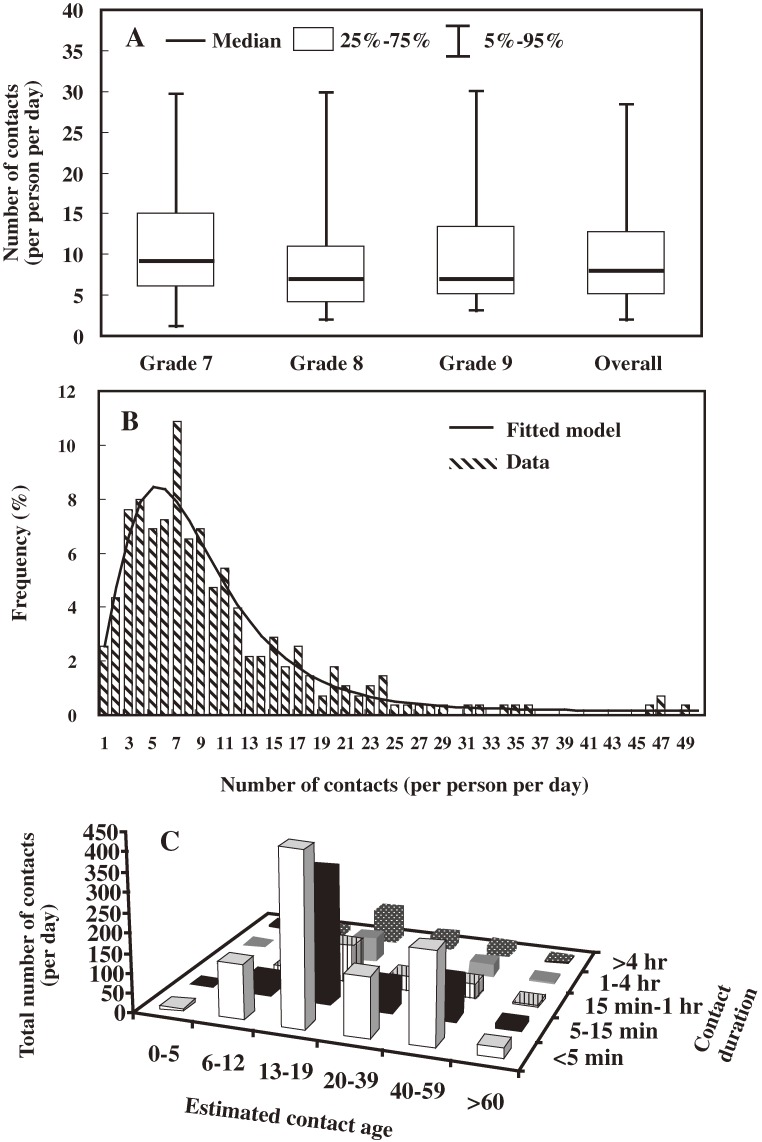
(A) Box-whisker plots showing the number of contacts per person per day (with 2.5% to 97.5% and 25% to 75% percentile distributions) among schoolchildren in grades 7 to 9. (B) Frequency distribution for number of contacts per person per day. (C) Total number of contacts per day in 6 estimated contact age groups (0–5, 6–12, 13–19, 20–39, 40–59, and >60 years) by contact duration (<5 minutes, 5–15 minutes, 15 minutes–1 hr, 1–4 hours, and >4 hours) in contact level 1.

The numbers and percentages of all participants were 41 (29.93%), 44 (32.12%), and 52 (37.96%) for grades 7 to 9, respectively. Figure 2A shows the box-whisker plot of number of contacts per person per day and the overall average for grades 7 to 9. The estimated median number (SD) of contacts per person per day for grades 7, 8, and 9 were 9.44 ± 8.68, 10.12 ± 4.5, and 11.18 ± 7.98, respectively. There was similar contact behavior across school grades. Figure 2B shows the frequency distributions for number of contacts per person per day, which included all contacts in grades 7 to 9. The results suggest skewness of contact behavior with a best-fit lognormal distribution (*R*^2^ = 0.94).

In the level 1 contact survey (2-way conversations with at least 3 spoken words), the relationships among total number of contacts per day in relation to estimated contact age group (0–5, 6–12, 13–19, 20–39, 40–59, and >60 years) and contact duration (<5 minutes, 5–15 minutes, 15 minutes–1 hour, 1–4 hours, and >4 hours) are shown in Figure 2C. A contact duration of less than 5 minutes was most prevalent in the age group 13 to 19 years, which included 436 contacts (representing 72% and 12% of contacts at school and home, respectively), and in the age group 40 to 59 years, which included 206 contacts (representing 11% and 84% at another location and home, respectively).

### Size-dependent droplet number concentrations for coughing and talking

**Figure 3.**
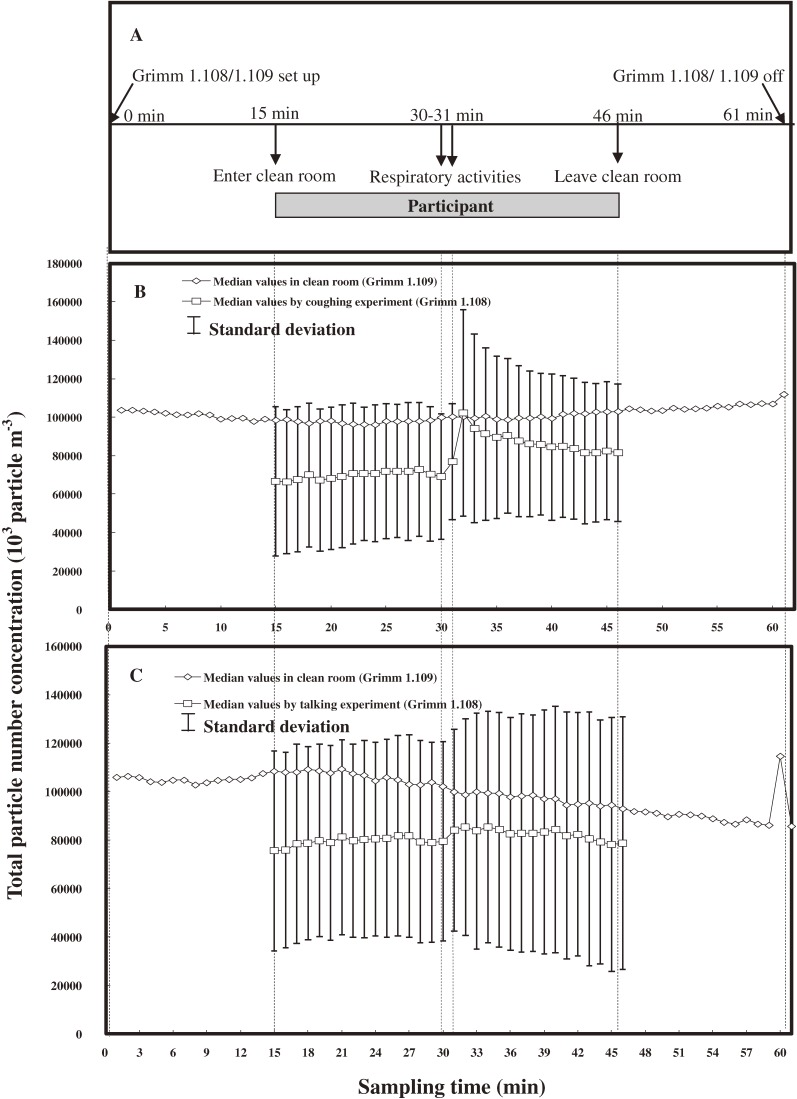
(A) Time-scale design for droplet experiments. The experimental design and data collection are detailed in the Methods. (B) The average median concentration number (for droplets >0.3 µm) for 10 participants is shown for coughing (20 times) and (C) talking (droplets were measured by having the participants count from 1 to 20).

Table 1 shows the basic characteristics of participants and the sampling environment conditions in the droplet experiment. Among the 10 participants, 5 women (F1–F5) and 5 men (M1–M5) were tested. Temperature and relative humidity ranged from 23°C to 27.5°C and 90% to 91% in all tests, respectively. The experimental timeline and design is illustrated in Figure 3A . Average background concentrations in the clean room and median concentration of droplets with a diameter greater than 0.30 µm in the sampling box are shown for coughing in Figure 3B and talking in Figure 3C. Time-dependent droplet concentration number was highest at 32 minutes for both coughing (1.02 × 10^8^ droplets m^−3^) and talking (8.52 × 10^7^ droplets m^−3^). A trend toward increasing droplet concentration was obvious for coughing when respiratory activities were tested.

**Table 1.** Environmental conditions and selected background characteristics of participants

Subject	Age (yr)	Health status	Number	Respiratory activity	Temperature (°C) (Start/End)	RH (%)^a^ (Start/End)
F1	24	Healthy	1	Coughing	27.5/25	91/90
			2	Talking	25/25	90/90
F2	21	Healthy	3	Coughing	26/26	91/91
			4	Talking	25/25	90/90
F3	22	Healthy	5	Coughing	25/24	90/90
			6	Talking	24/24	90/90
F4	30	Influenza	7	Coughing	26/25	91/90
			8	Talking	27/25	91/90
F5	21	Influenza	9	Coughing	26/25	91/90
			10	Talking	25/25	90/90
M1	21	Healthy	11	Coughing	24.5/25	90/90
			12	Talking	25/25	90/90
M2	20	Healthy	13	Coughing	26/25	91/90
			14	Talking	26/25	91/90
M3	21	Healthy	15	Coughing	24/24	90/90
			16	Talking	24/25	90/90
M4	28	Influenza	17	Coughing	25/25	90/90
			18	Talking	25/25	90/90
M5	23	Influenza	19	Coughing	25/25	90/90
			20	Talking	23/23	90/90

^a^Relative humidity was determined by referring to a psychrometric chart after calculating the difference between wet bulb and dry bulb temperature.

The estimated size-dependent droplet concentration numbers for coughing and talking at 30 to 31 minutes is shown in Tables 2 and 3. Small-diameter (0.3–0.4 µm) droplets were more numerous than large-diameter (>4 µm) droplets. The total median droplet concentration numbers were 9.01 × 10^7^ and 8.23 × 10^7^ droplets m^−3^ for coughing and talking, respectively. Thus, talking clearly contributes to time-dependent droplet concentrations for coughing.

**Table 2.** Estimated median total droplet concentration, for different droplet diameter ranges, after coughing (20 times) at 30–31 min among female (F) and male (M) participants

Size distribution (µm)	Coughing (10^3^ droplets m^−3^)												

	F1	F2	F3	F4^a^	F5^a^	Median	M1	M2	M3	M4^a^	M5^a^	Median	Overall Median
0.3–0.4	39 164	113 911	124 274	57 516	52 459	57 516	52 834	84 336	82 883	77 684	50 391	77 684	67 600
0.4–0.5	9594	39 297	36 874	12 076	15 778	15 778	9772	17 896	28 955	26 904	16 485	17 896	17 190
0.5–0.65	1708	5522	7112	1605	4460	4460	1315	2684	4955	5428	3999	3999	4229
0.65–0.8	278	403	620	320	770	403	303	585	470	693	628	585	528
0.8–1	160	128	228	158	423	160	163	305	305	393	303	305	265
1–1.6	80	58	105	40	183	80	103	185	153	188	153	153	129
1.6–2	36	15	33	68	98	36	32	138	101	118	86	101	77
2–3	36	19	43	35	89	36	19	147	85	105	72	85	58
3–4	7	9	7	2	13	7	2	26	12	9	10	10	9
4–5	1	2	3	1	5	2	1	13	3	3	5	3	3
5–7.5	2	3	3	2	1	2	0	7	2	1	3	2	2
7.5–10	1	1	4	1	0	1	0	1	1	0	1	1	1
10–15	0	1	5	0	1	1	0	0	0	0	0	0	0
15–20	0	0	0	0	0	0	0	0	0	0	0	0	0
>20	0	0	0	0	0	0	0	0	0	0	0	0	0
Total	51 065	159 364	169 310	71 822	74 277	78 479	64 540	106 320	117 922	111 522	72 133	100 821	90 088

^a^F4, F5, M4, and M5 were influenza patients.

**Table 3.** Estimated median total droplet concentration, for different droplet diameter ranges, after talking (counting from 1 to 20) at 30–31 min among female (F) and male (M) participants

Size distribution (µm)	Talking (10^3^ droplets m^−3^)												

	F1	F2	F3	F4^a^	F5^a^	Median	M1	M2	M3	M4^a^	M5^a^	Median	Overall Median
0.3–0.4	16 067	93 488	83 179	38 960	83 181	83 179	23 547	21 533	78 988	89 729	45 174	45 174	62 081
0.4–0.5	2910	31 211	19 430	5881	19 368	19 368	4396	3973	23 325	29 002	14 051	14 051	16 710
0.5–0.65	823	4634	2679	925	2869	2679	763	728	3749	4452	3920	3749	2774
0.65–0.8	300	398	370	238	505	370	163	168	448	533	890	448	384
0.8–1	208	140	278	148	293	208	100	115	253	223	610	223	215
1–1.6	88	58	103	53	123	88	13	50	110	105	305	105	95
1.6–2	48	50	80	35	74	50	11	39	50	50	166	50	50
2–3	26	8	47	15	38	26	6	16	32	37	155	32	29
3–4	5	2	4	1	4	4	0	3	6	2	37	3	3
4–5	1	2	2	1	2	2	1	1	0	2	9	1	1
5–7.5	0	0	1	1	1	1	0	0	0	0	4	0	0
7.5–10	0	1	0	1	0	0	0	0	0	0	1	0	0
10–15	1	1	1	1	0	1	0	0	0	0	0	0	0
15–20	0	0	0	0	0	0	0	0	0	0	0	0	0
>20	0	0	0	0	0	0	0	0	0	0	0	0	0
Total	20 474	129 990	106 170	46 255	106 455	105 972	28 997	26 623	106 958	124 132	65 319	63 833	82 340

^a^F4, F5, M4, and M5 were influenza patients.

### Large-droplet concentration on slides

**Figure 4.**
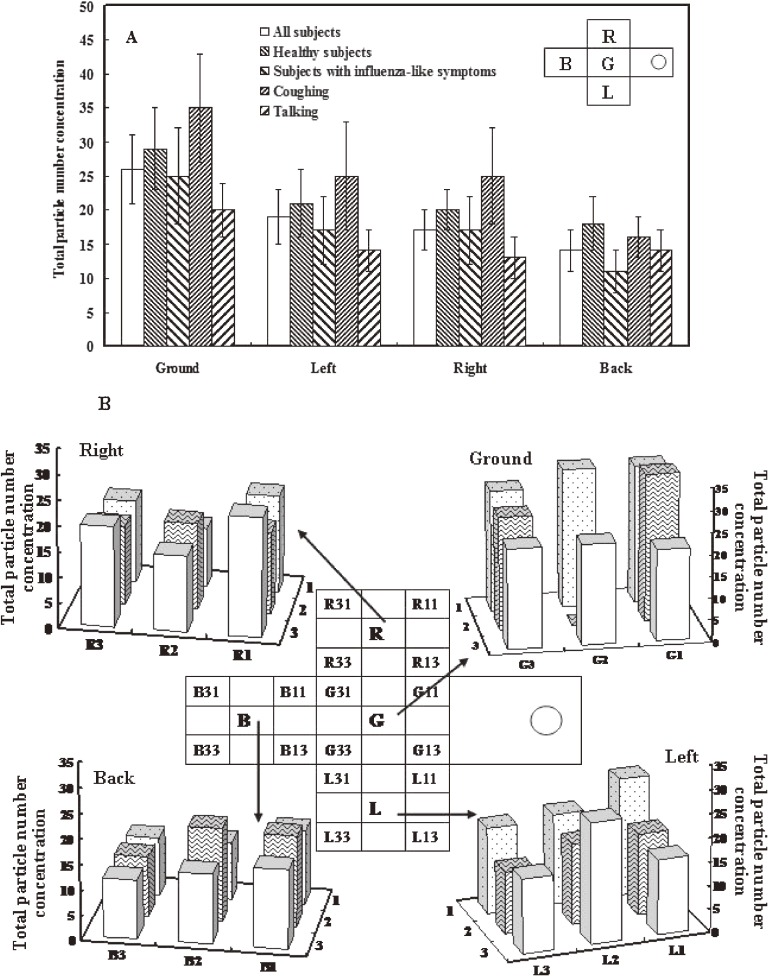
(A) Total droplet concentration numbers (mean ± SD) on the floor and left, right, and back walls of the sampling box for all subjects (*N* = 10), healthy subjects (*N* = 6), subjects with influenza-like symptoms (*N* = 4), subjects in the coughing experiment (*N* = 10), and subjects in the talking experiment (*N* = 10). (B) Relative position of numbers on glass slides and total droplet concentration numbers for the 4 walls of the sampling box.

Figure 4 shows droplet concentration number on the 4 inside walls of the sampling box as observed on glass slides with a microscope. The estimated mean (SD) total droplet concentration numbers were 26 ± 5, 19 ± 4, 17 ± 3, and 14 ± 3 droplets on the floor, left, right, and back slides, respectively, indicating that the droplets were easily deposited on the box floor (Figure 4A). For coughing, the estimates were 35 ± 8 (mean ± SD) and 16 ± 3 droplets on the floor and back slides, whereas for talking the estimates were 20 ± 4 and 14 ± 3 droplets on the floor and back slides. The number of large droplets generated was clearly higher for coughing than for talking. We also investigated individual droplet concentration for different slide positions in all participants (Figure 4B). The deposition pattern positively correlated with distance of the slide from the hole and slightly negatively correlated with droplet concentration.

### Schoolchildren population dynamics

**Figure 5.**
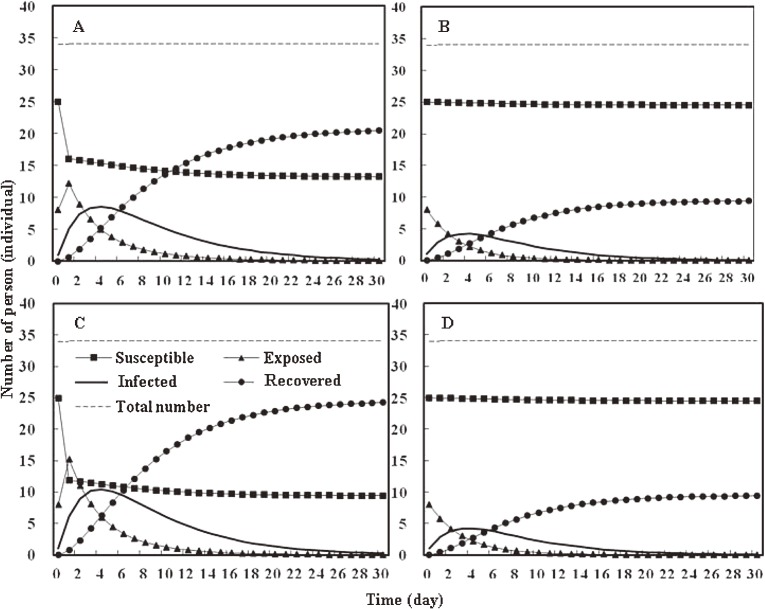
Population dynamic modeling of schoolchildren (*N* = 34), using Eqs. (4) to (9) and the parameters listed in Table 4. Modeling results of human talking activity by size-dependent median number of droplets per individual participant at (A) *D*_d1_ = 8.23 × 10^7^ and (B) *D*_d2_ = 1.00 × 10^3^. Results of model of human coughing activity by size-dependent median number of droplets per individual participant at (C) *D*_d1_ = 9.01 × 10^7^, and (D) *D*_d2_ = 5 × 10^3^.

Table 4 lists the parameters used for transmission rate estimation and SEIR modeling. The transmission rates, *β* in Eq. (3), were estimated to be 3.35 × 10^−7^ and 3.29 × 10^−6^ day^−1^ for *d*_1_ (4 µm) and *d*_2_ (4–8 µm) by calculating the product of $\tilde{C}$ = 7.8 × 10^−3^ and either ${\tilde{P}}_{d}$ = 4.19 × 10^−5^ or ${\tilde{P}}_{d}$ = 4.22 × 10^−4^, respectively. The results showed that size-dependent transmission rate differed by a factor of 10. We incorporated the parameter values (Table 4) into Eqs. (4) to (9) to estimate the indoor transmission dynamics of influenza infection among schoolchildren. Figure 5 shows the results of modeling human talking (A–B) and coughing (C–D) activities for different size-dependent droplets. For the talking activity, the size-dependent median number of droplets per participant, *D*_d1_ = 8.23 × 10^7^ and *D*_d2_ = 1.00 × 10^3^, caused a maximum of 8 infected persons at day 4 (Figure 5A) and 4 infected persons at day 3 (Figure 5B), respectively. For the coughing activity, the size-dependent median number of droplets per participant, *D*_d1_ = 9.01 × 10^7^ and *D*_d2_ = 5 × 10^3^, caused a maximum of 10 infected persons at day 4 (Figure 5C) and 4 infected persons at day 4 (Figure 5D), respectively. These results imply that the flu can spread in classrooms. We believe that the difference may due in part to small droplet sizes, which could result in a greater number of suspended droplets in air.

**Table 4.** Parameters used for transmission rate estimation and susceptible–exposed–infected–recovery (SEIR) modeling

Parameter	Description	Value	Reference
**Transmission rate estimation**			
*c*	Average number of susceptible contacts with any individual	10.15 (day^−1^)	22
*B*	Inhalation rate	20.1 (m^3^ day^−1^)	31, 34
*τ*	Duration of contact between susceptible and infected	1.04 × 10^−2^ (day)	17
*V*_cl_	Personally generated droplet volume	8.0 m^3^	2
*P*_d_	Probability of transmission spread with susceptible per inhaled pathogen-carrying droplet	0.052	32, 35
*q*_dep_	Probability of deposition in human respiratory tract per pathogen	*q*_dep,d1_ = 0.88 *q*_dep,d2_ = 0.99	2, 36
*N*_d_	Number of pathogens in droplet of given diameter	*N*_d1_ = 9.15 × 10^−4^ *N*_d2_ = 8.2 × 10^−3^	2, 36
**SEIR model**			
*σ*	Rate at which an exposed person becomes an infectious person per unit of time	0.333 (day^−1^)	23
*γ*	Rate of recovery for infected person	0.2 (day^−1^)	24
*k*	Droplet production rate	*k*_d1_ = 4.10 × 10^5^ (day^−1^) *k*_d2_ = 1.92 × 10^4^ (day^−1^)	28
*ν*	Droplet removal rate	*ν*_d1_ = 6.83 × 10 (day^−1^) *ν*_d2_ = 1.56 × 10^2^ (day^−1^)	33, 37
*D*_0_	Median number of droplets per participant at 1 time	For talking: *D*_d1_ = 8.23 × 10^7^ and *D*_d2_ = 1 × 10^3^ For coughing: *D*_d1_ = 9.01 × 10^7^ and *D*_d2_ = 5 × 10^3^	Estimated from droplet experiment

^a^*d*_1_ = 4 µm, *d*_2_ = 4–8 µm.

## DISCUSSION

To investigate the dynamics of indoor transmission, we used a disease population transmission model that linked contact rates and droplet concentration estimates with respiratory infections. For the questionnaire analyses, the overall mean (SD) number of contacts was 5.66 ± 6.23 day^−1^, with a min–max range of 0–44 day^−1^ in the age group 13 to 19 years. In contrast, the mean number of contacts was 1.96 ± 2.76 day^−1^, with a min–max range of 0–29 day^−1^ in the age group 40 to 59 years. McCaw et al^15^ found that, for all encounters, the number of observations within each age group varied between 1 day^−1^ (in the age group 70–79 years) and 22 day^−1^ (in the age group 40–49 years). Mikolajczyk et al^19^ also used questionnaires in a primary school in Germany. The mean number of contacts was 25.1 ± 16.5 day^−1^ (min–max range, 0–78) in children and 7.5 ± 5.0 day^−1^ (min–max range, 1–47) in adults. Questionnaire surveys of social contact characteristics were highly structured according to age.

We believe that a 67% response rate (*N* = 404) for our questionnaire survey is acceptable for the present analyses. Mikolajczyk et al^19^ and Beutels et al^14^ reported questionnaire response rates of 79.4% (*N* = 296) and 69.8% (*N* = 73), respectively. The lower response rate in our study might be due in part to the 1-week delay in questionnaire replies. Recovery percentage might be improved by providing incentives or increasing the survey frequency of the same population. Unfortunately, our questionnaire results could not be used to quantify the severity of symptoms, due to the lack of physicians.

There were 10 participants (5 women and 5 men) in the droplet experiment: 6 healthy participants and 4 with influenza-like symptoms. Yang et al^25^ found that the difference in average droplet size was not significant (*P* > 0.1) between males (*N* = 27) and females (*N* = 27); however, average droplet concentration significantly differed (*P* < 0.1) by sex. In our study, men had higher droplet concentrations in the coughing experiment. Thus, our results agree with those of Yang et al.^25^

Droplet concentration was higher after coughing than after talking, which is consistent with previous studies.^3^^,^^7^^,^^8^ However, the environmental concentration estimated by the Grimm 1.109 was unstable in the clean room, suggesting that this value was influenced by door opening/closing or other human activities. The clean room was connected to the central air conditioning system; hence, ventilation rate could not be estimated in our experimental design. Furthermore, the 15-minute time span alloted for droplet deposition might need to be increased. Coughing and talking have different biologic mechanisms, and mouth/nose opening sizes and initial velocity also varied, which may have affected the results. Therefore, exhaled airflow and related factors should be studied next.

Factors other than the presently investigated activities and frequency of experimentation are likely to be important. Gralton et al^6^ reviewed the role of droplet size in aerosolized pathogen transmission and found that factors affecting disease transmission included type of respiratory activity, frequency of respiratory activity, site of infection, pathogen load, and pathogen type. Fabian et al^10^ and Lindsley et al^26^ also measured airborne influenza virus in aerosol droplets from human coughs.

As for other factors, Tellier^27^ reviewed studies of aerosol transmission of influenza A virus and found that temperature, relative humidity, and ventilation might influence exhaled droplet concentrations in a sampling box. Gralton et al^6^ also reported factors that determined how droplets facilitate aerosolized transmission, including relative humidity, aggregation, pre-exposure of airways to saline, and disease state. In the present study, the ranges for temperature and relative humidity were 23°C to 27.5°C and 90% to 91%, respectively. Relative humidity may affect evaporation rate and droplet equilibrium size.^28^

Although difficult, it is essential to validate the parameters and proposed model with the real world. Traditional models (such as the susceptible–infected–recovery model) are limited in their ability to analyze the relationship between the mechanism of inhalation infection and human behavior, because they only explore contact-related transmission rate. We believe that the parameters of *c* = 10.15 day^−1^ and *τ* = 1.04 × 10^−2^ day were acceptable estimations, based on data from age-specific questionnaire surveys of contact rates.^1^^,^^13^^–^^17^^,^^19^^,^^22^ As for the range of individual infection, important factors include time that pathogen-laden droplets are suspended in air, humidity, environmental temperature, and individual immunity. Therefore, some recent studies have used personal droplet volume, *V*_cl_, to precisely estimate personal exposure to surrounding air.^2^^,^^29^^,^^30^ However, exposing humans to respiratory infection in experiments has potential risks, and such transmission is difficult to measure between humans. Future research should seek to improve the technology used to immediately detect droplet-laden pathogen transmission between humans and to develop a robust model of such transmission.

Regarding parameter estimates, transmission rate is the most important in flu spread. On the basis of Eqs. (1) to (3), parameters such as *c* (average number of susceptible contacts with any individual [day^−1^] in unit of time *δt*), *τ* (duration of contact between susceptible and infected [day]), and *N*_d_ (number of pathogens in a droplet) cannot be immediately measured. Hence, it is difficult to estimate many parameters. The parameter *γ*, ie, the rate at which an infectious individual recovers per unit time, is also difficult to determine. We did not consider flu severity among children in the SEIR model. Recovery duration may be affected by differences between adults and children in immunity, nutritional status, and socioeconomic characteristics. Finally, because most participants in the respiratory activity experiments were healthy, droplet concentrations might have been underestimated in those experiments.

The SEIR model uses a closed population to simulate both infection in an individual over time and subsequent development of immunity.^31^ Findings from recent studies are consistent with those of the present study.^2^^,^^32^ Noakes et al^33^ used a SEIR model to simulate indoor environments and found that the model could combine infection characteristics and the physical environment. Hence, our results suggest that flu spreads in classrooms and that infected persons ultimately recover.

Our study does have some limitations. First, loudness and pitch during talking or coughing could not be controlled in the droplet experiment. Second, in the modeling of population dynamics among schoolchildren, it would have been better to have used the same representative population that was studied in the questionnaire survey and droplet experiment. Third, this study used microscope evaluation of glass slides to measure droplet numbers in a sampling box. However, droplet size and distribution could not be estimated. Thus, we could not incorporate these data into the mathematical model. Finally, by incorporating daily contact numbers and droplet concentration into the SEIR model, we showed that droplet diameter was related to infectious potential.

In conclusion, by linking a classical compartmental epidemiologic model with a behavioral transmission model we explored the impact of contact behavior and exhaled droplet patterns on indoor epidemiologic processes among schoolchildren. Our results suggest that human contact behavior and airborne droplet characteristics significantly alter the predicted indoor population transmission dynamics of influenza infection. This mechanistic understanding may aid in investigating potential control measures or interventions, such as personal protection masks or social distance. Such measures could alter contact behavior and droplet characteristics, minimize exhalation of infectious droplets, and ultimately reduce infection risk in indoor environments.
